# Brain morphological and connectivity changes on MRI after stem cell therapy in a rat stroke model

**DOI:** 10.1371/journal.pone.0246817

**Published:** 2021-02-16

**Authors:** Jeong Pyo Son, Ji Hee Sung, Dong Hee Kim, Yeon Hee Cho, Suk Jae Kim, Jong-Won Chung, Won Hyuk Chang, Yun-Hee Kim, Eun Hee Kim, Gyeong Joon Moon, Oh Young Bang

**Affiliations:** 1 Department of Health Sciences and Technology, Samsung Advanced Institute for Health Sciences and Technology (SAIHST), Sungkyunkwan University, Seoul, South Korea; 2 Translational and Stem Cell Research Laboratory on Stroke, Samsung Medical Center, Seoul, South Korea; 3 Laboratory Animal Center, Osong Medical Innovation Foundation, Osong, South Korea; 4 S&E bio, Inc. Seoul, South Korea; 5 Department of Neurology, Samsung Medical Center, Sungkyunkwan University School of Medicine, Seoul, South Korea; 6 Physical and Rehabilitation Medicine, Samsung Medical Center, Sungkyunkwan University School of Medicine, Seoul, South Korea; 7 Stem Cell and Regenerative Medicine Institute, Samsung Medical Center, Seoul, South Korea; 8 AMC Stem Cell Center, Asan Medical Center, Seoul, South Korea; Henry Ford Health System, UNITED STATES

## Abstract

In animal models of stroke, behavioral assessments could be complemented by a variety of neuroimaging studies to correlate them with recovery and better understand mechanisms of improvement after stem cell therapy. We evaluated morphological and connectivity changes after treatment with human mesenchymal stem cells (hMSCs) in a rat stroke model, through quantitative measurement of *T*_2_-weighted images and diffusion tensor imaging (DTI). Transient middle cerebral artery occlusion rats randomly received PBS (PBS-only), FBS cultured hMSCs (FBS-hMSCs), or stroke patients’ serum cultured hMSCs (SS-hMSCs). Functional improvement was assessed using a modified neurological severity score (mNSS). Quantitative analyses of *T*_2_-weighted ischemic lesion and ventricular volume changes were performed. Brain microstructure/connectivity changes were evaluated in the ischemic recovery area by DTI-derived microstructural indices such as relative fractional anisotropy (rFA), relative axial diffusivity (rAD), and relative radial diffusivity (rRD), and relative fiber density (rFD) analyses. According to mNSS results, the SS-hMSCs group showed the most prominent functional improvement. Infarct lesion volume of the SS-hMSCs group was significantly decreased at 2 weeks when compared to the PBS-only groups, but there were no differences between the FBS-hMSCs and SS-hMSCs groups. Brain atrophy was significantly decreased in the SS-hMSCs group compared to the other groups. In DTI, rFA and rFD values were significantly higher and rRD value was significant lower in the SS-hMSCs group and these microstructure/connectivity changes were correlated with *T*_2_-weighted morphological changes. *T*_2_-weighted volume alterations (ischemic lesion and brain atrophy), and DTI microstructural indices and rFD changes, were well matched with the results of behavioral assessment. These quantitative MRI measurements could be potential outcome predictors of functional recovery after treatment with stem cells for stroke.

## Introduction

Although most preclinical studies show promising results for stem cell therapy in various small animal models of stroke, randomized trials of stem cell therapy in stroke patients show negative or mixed results [1–8]. A systematic search for reports of experiments using stem cells in animal models of stroke showed that while stem cells appear to be of some benefit in animal models of stroke, the validity of preclinical data is potentially confounded by poor study quality, lack of standards in the conducting and reporting of experiments, publication bias, and significant experimental design differences between clinical and preclinical trials [9–11].

In addition, such discrepancy between preclinical and clinical studies calls for the need for objective parameters to evaluate the effects of stem cells in small animal models of stroke. In animal models of stroke, behavioral assessments could be complemented by a variety of neuroimaging studies to identify correlations with recovery after stroke [12–14], and thus better understand mechanisms of improvement. Recently, there have been growing efforts to visualize the microstructure and anatomical connectivity of the brain, using noninvasive imaging methods [15, 16]. Most importantly, modern magnetic resonance imaging (MRI) techniques such as diffusion tensor imaging (DTI) play important roles in both clinical and preclinical research. Diffusion tensor imaging is able to visualize microstructural alterations in various neurological disorders [17–21]. Moreover, recent experimental studies in rodent stroke models have shown that quantitative changes in diffusion tensor tractography and DTI indices, such as fractional anisotropy (FA), axial diffusivity (AD), and radial diffusivity (RD), can detect changes in axonal integrity and remyelination [12–14, 22, 23]. Therefore, imaging-based biomarkers can be good tools to investigate the therapeutic efficacy of stem cell therapy in ischemic stroke.

We aimed to evaluate morphological and connectivity changes after stem cell therapy in an animal model of stroke. A randomized controlled trial of stem cell therapy for ischemic stroke patients (STem Cell Application Researches and Trials in NeuroloGy [STARTING]-2 trial. Clinicaltrial.gov@indentifier NCT01716481) is currently evaluating the objective improvement and underlying mechanisms of recovery using multimodal MRI features, including DTI [8, 24]. Comparison of the effects of stem cell therapy, in both preclinical and clinical studies using the same MRI measurements, would be valuable. To this end, we evaluated MRI measurements (including *T*_2_-weighted MRI for ischemic lesion and ventricular volume changes, and DTI for microstructural indices and fiber density changes) after intravenous injection of human mesenchymal stem cells (hMSCs), either naïve or preconditioned, in a rat transient middle cerebral artery occlusion (tMCAo) stroke model.

## Materials and methods

All human subject research was approved by the Institutional Review Board (*Samsung Medical Center Institutional Review Board*, *Approval No*. *SMC 2011–10-047-047*). All patients or guardians of patients provided written informed consent to participate in this study. All animal experimental procedures were approved by Institutional Animal Care and Use Committee (*Laboratory Animal Research Center; AAALAC International approved facility*, *Approval No*. *001003*) of the Samsung Medical Center and performed in accordance with the Animal Research: Reporting of In Vivo Experiments (ARRIVE) guidelines [25]. All animals were kept under the conditions of a temperature of 22±1 °C, a relative humidity of 50±10%, a lighting time of 12 hours (8 a.m. to 8 p.m.), a ventilation frequency of 10 to 20 times/hr, and an illuminance of 150 to 300 Lux. All animals were observed twice daily for health monitoring and no adverse events occurred. A completed ARRIVE guidelines checklist is included in S1 Checklist.

### Focal cerebral ischemia model

Adult male Sprague-Dawley rats (Orient Bio Inc., Seongnam, South Korea) weighing 250 to 300 g (7–8 weeks old) were used in this study. Rats were induced with tMCAo as previously described [26]. Briefly, anesthesia was delivered using a face mask with 5% isoflurane, and maintained with 2.0% isoflurane in 70% N_2_O and 30% O_2_. During the surgical procedure, body temperature was maintained at 37.0 °C to 37.5 °C (measured rectally). The right common carotid artery (CCA), external carotid artery, and internal carotid artery were loosely ligated with a 4–0 silk suture. The right middle cerebral artery (MCA) was occluded with a silicon-coated nylon suture (tip diameter: 330–380 mm) from the right CCA. After 90 minutes of occlusion, reperfusion was performed by removing the suture.

### Preparation of serum and mesenchymal stem cells

Serum was collected from ischemic stroke patients enrolled in the STARTING-2 trial within 90 days after onset of stroke (n = 9, 30.4 ± 18.1 day) [24]. Single aliquots of serum were stored at –70 °C until further use. Patient basal characteristics were previously reported [26].

Passage 2 hMSCs at were purchased from Lonza, Basel, Switzerland (Cat No. PT-2501) and grown in Dulbecco’s modified Eagle’s medium (DMEM, Invitrogen, Carlsbad, CA, USA) with 10% fetal bovine serum (FBS; Hyclone, Victoria, Australia) for the first passage. The medium was then replaced with 10% FBS or 10% stroke patients’ serum (SS). Passage 5 hMSCs (2 x 10^6^ cells) were transplanted to study their therapeutic effects [26].

### Experimental groups and intravenous infusion of hMSCs

In this study, a total of 45 animals were randomly grouped according to treatment: intravenous infusion of hMSCs (2 x 10^6^ cells in 1 mL total fluid volume) cultured with ischemic stroke patients’ serum (SS-hMSCs group); intravenous infusion of hMSCs cultured with FBS (FBS-hMSCs group); intravenous infusion of phosphate-buffered saline (PBS-only group). The suspended hMSCs or PBS were slowly administered one day after tMCAo (immediately after the first MRI session) via tail vein of the rat. Four animals died within 24 h after stroke were excluded (SS-hMSCs, n = 2; FBS-hMSCs, n = 1; PBS-only, n = 1). Four animals without observable neurological deficits were excluded (SS-hMSCs, n = 1; FBS-hMSCs, n = 1; PBS-only, n = 2). Two animals with subarachnoid hemorrhage were excluded (SS-hMSCs, n = 1; FBS-hMSCs, n = 1). A total of 35 animals were included in the final analysis (SS-hMSCs group, n = 5 for MRI and n = 6 for behavioral testing; FBS-hMSCs group, n = 6 for MRI and n = 6 for behavioral testing; PBS-only group, n = 6 for MRI and n = 6 for behavioral testing).

### MRI image acquisition

MRI was performed using a 7T small animal MR scanner (Bruker Biospin 70/20 USR, Fällanden, Switzerland). A quadrature birdcage coil (inner diameter = 72 mm) was used for excitation and an actively decoupled 4-channel phased array surface coil was used for receiving the signal. *T*_2_-weighted and DTI, were acquired 1 day, 2 weeks and 5 weeks after stroke onset, under isoflurane anesthesia (5% for induction, 1.5% for maintenance).

*T*_2_-weighted images were acquired using a turbo rapid acquisition with refocusing echoes (Turbo RARE) sequence with the following parameters: repetition time (TR)/echo time (TE) = 3000/60 ms, field of view = 30 x 30 mm^2^, image matrix = 192 x 192, and in-plane resolution = 0.156 x 0.156 x 0.75 mm^3^.

DTI images were acquired using a 4 shot spin-echo echo planar imaging sequence (Stejskal-Tanner pulsed-gradient sequence) with the following parameters: TR/TE = 4500/45 ms, field of view = 30 x 30 mm^2^, image matrix = 192 x 192, in-plane resolution = 0.156 x 0.156 x 0.75 mm^3^, diffusion encoding gradient directions = 30, gradient duration (δ) = 5 ms, gradient separations (Δ) = 15 ms, and b-value = 1000 s/mm^2^.

### MRI image analysis

All MRI images were analyzed blinded to the experimental group information. Analyses of *T*_2_-weighted images were performed to estimate the ischemic lesion area and lateral ventricular volume. The infarct lesion area on each *T*_2_-weighted image slice was identified by those voxels with a *T*_2_ value higher than the mean + two standard deviations (mean + 2SD) of normal tissue in the contralateral hemisphere [14, 23]. Lateral ventricular volume was delineated from 8 contiguous *T*_2_-weighted images with reference to Paxinos stereotaxic rat brain atlas [27]. Ischemic lesion and lateral ventricle volumes were normalized to the volume at day 1, to compensate for individual bias.

Analysis of DTI data was performed to observe the microstructural reorganization of ischemic lesions using FMRIB’s Diffusion Toolbox (FSL, http://fsl.fmrib.ox.ac.uk/fsl), and DSI Studio (http://dsi-studio.labsolver.org). Raw DTI images were pre-processed, using the following procedures: 1) separate brain from non-brain structures and create a brain mask image using the FSL’s brain extraction tool (BET), 2) realign diffusion weighted images to the non-diffusion weighted b0 image for correction of artifacts due to head motion and eddy current distortions, using the FSL’s Eddycorrect tool, and 3) calculate diffusion tensor at each voxel and create a DTI index map, such as FA, AD, and RD, using the FSL’s DTIFIT tool. After calculation of diffusion tensor, fiber tracking using a generalized deterministic algorithm was performed using DSI studio [28]. Fiber tracking was terminated at locations where the FA value fell below 0.15 or the turning angle was greater than 45 degrees. Fiber tractography results were analyzed using fiber density (FD), which was calculated by dividing the total number of reconstructed fiber tracts by the number of seeding voxels.

**Fig 1:**
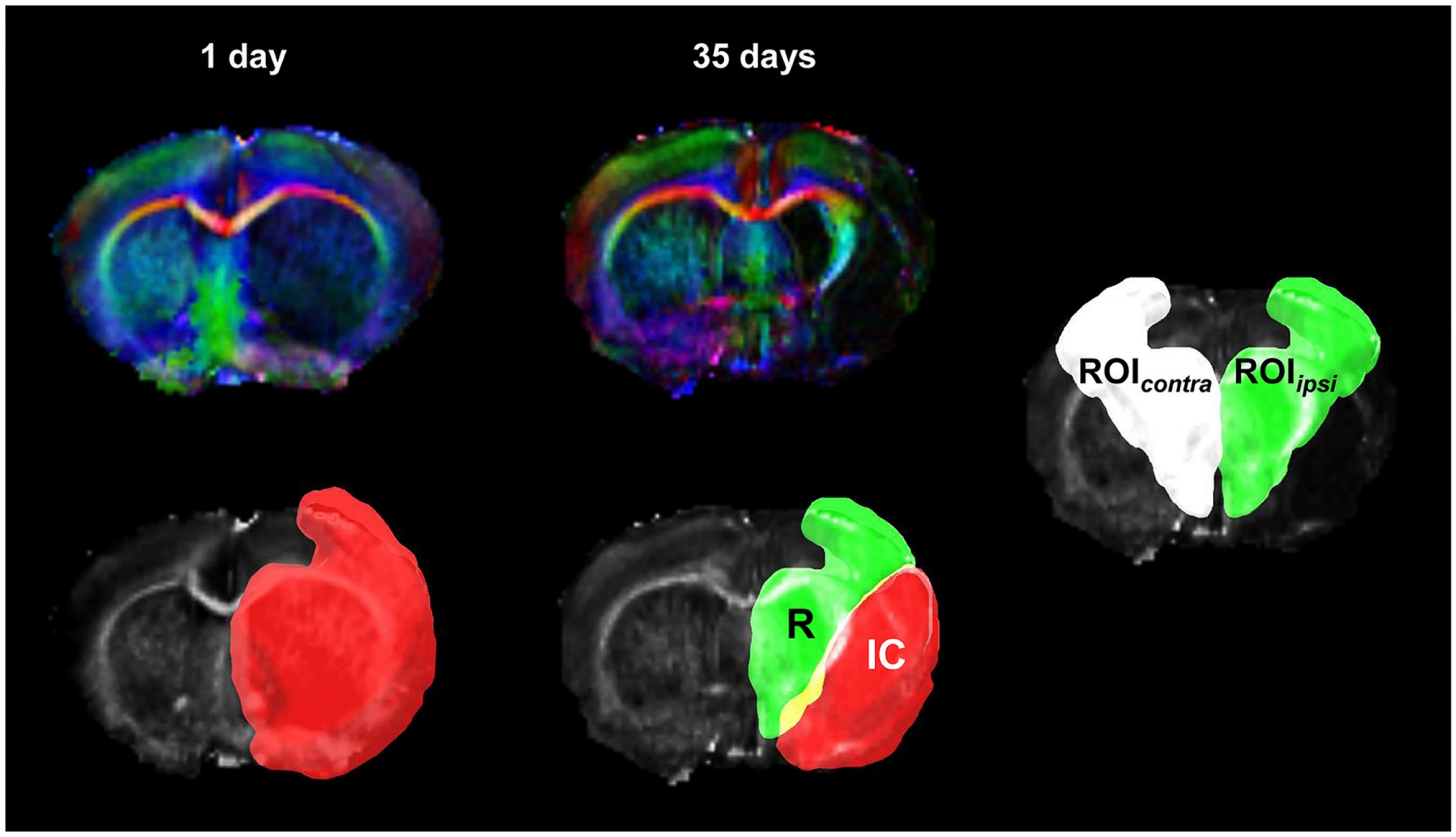
Ipsilesional region of interest (ROI) was defined by subtracting the ischemic damaged regions at 5 weeks after ischemia from those at day 1. Mirror ROI was defined as our contralesional ROI.

For quantification of microstructural indices and FD values, a region of interest (ROI) was selected in the ischemic recovery area on *T*_2_-weighted images at 5 weeks after stroke onset [22, 23]. The ischemic damaged area was delineated by the mean, plus twice the standard deviation of *T*_2_-weighted signal intensity of the contralateral side, at 1 day and 5 weeks after experimental stroke. In *T*_2_-weighted images, the ischemic damaged areas on day 1 were consistently larger than those at 5 weeks after stroke. Thus, the recovery area was defined as our ROI, by subtracting the ischemic damaged areas at 5 weeks from the ischemic damaged area, 1 day after stroke (Fig 1). Contralateral homologous ROIs were also defined in the contralateral side. Relative changes in FA (rFA), AD (rAD), RD (rRD), and FD (rFD) values were calculated by dividing the values of ipsilesional ROI by those of contralesional ROI to compensate for individual variation.

### Behavioral testing

In all animals, behavioral tests were performed before tMCAo, and at 1 day, 2 weeks and 5 weeks after tMCAo, by an investigator who was blind to the experimental groups. Modified Neurological Severity Scores (mNSS) were calculated as a measure of motor and sensory reflex functions, and balance using modified versions of sensory tests, as previously described [26, 29]. The total mNSS was scaled from 0 to 18 (normal = 0, maximal deficit = 18) and determined by measuring responses to being raised by the tail (subtotal score = 0 to 3: forelimb flexion = 0 to 1, hindlimb flexion = 0 to 1, and head movement over 101 degrees to the vertical axis within 30 s = 0 to 1), the results of sensory tests (subtotal score = 0 to 12: visual placement of forelimbs = 0 to 3, tactile placement of forelimbs = 0 to 3, proprioceptive adduction of hindlimbs = 0 to 3, tactile placement of hindlimbs = 0 to 3), and the results of beam balance tests (subtotal score = 0 to 6: balances with steady posture = 0, grasps side of beam = 1, hugs beam and one limb falls down = 2, hugs beam and two limbs fall down or spins after 60 s = 3, attempts to balance on beam but falls off after 40 s = 4, attempts to balance on beam but falls off after 20 s = 5, falls off and makes no attempt to balance or hang onto beam within 20 s = 6).

### Immunostaining

To visualize myelinated fiber in the ischemic damaged area, immunostaining was performed. Animals were euthanized and transcardially perfused with PBS and 4% paraformaldehyde (PFA) immediately after the final MRI scanning at 5 weeks after treatment. The paraffin-embedded brains were sectioned coronally between 3 and 4 mm posterior to the bregma to a thickness of 18 μm in accordance with the MRI images using a Cryocut Microtome (Leica Microsystems). Glass mounted, 18 μm brain sections were stained with luxol fast blue (LFB) after deparaffinization and rehydration.

### Statistical analysis

All data are presented as mean ± SD. To evaluate the interaction between treatment effect and time, a generalized estimating equation (GEE) model was used [14]. Statistical analysis between treatment groups at each time period were performed using one-way analysis of variance (ANOVA) followed by Tukey post hoc analysis only when the *p*-value of GEE model test attained < 0.05. To analyze the relationship between morphological changes in *T*_2_-weighted imaging and DTI results, Pearson correlation analysis was performed. Correlation analysis results are expressed as Pearson correlation coefficients (r) and *p* values. *P* values<0.05 were considered statistically significant. Statistical analyses were performed using the commercially available software package, SPSS version 18 (SPSS Inc., Chicago, IL, USA). Graphs were drawn using Graph Pad Prism 8 (Graph Pad Software Inc., San Diego, CA, USA).

## Results

### Functional improvement after MSC treatment

**Fig 2:**
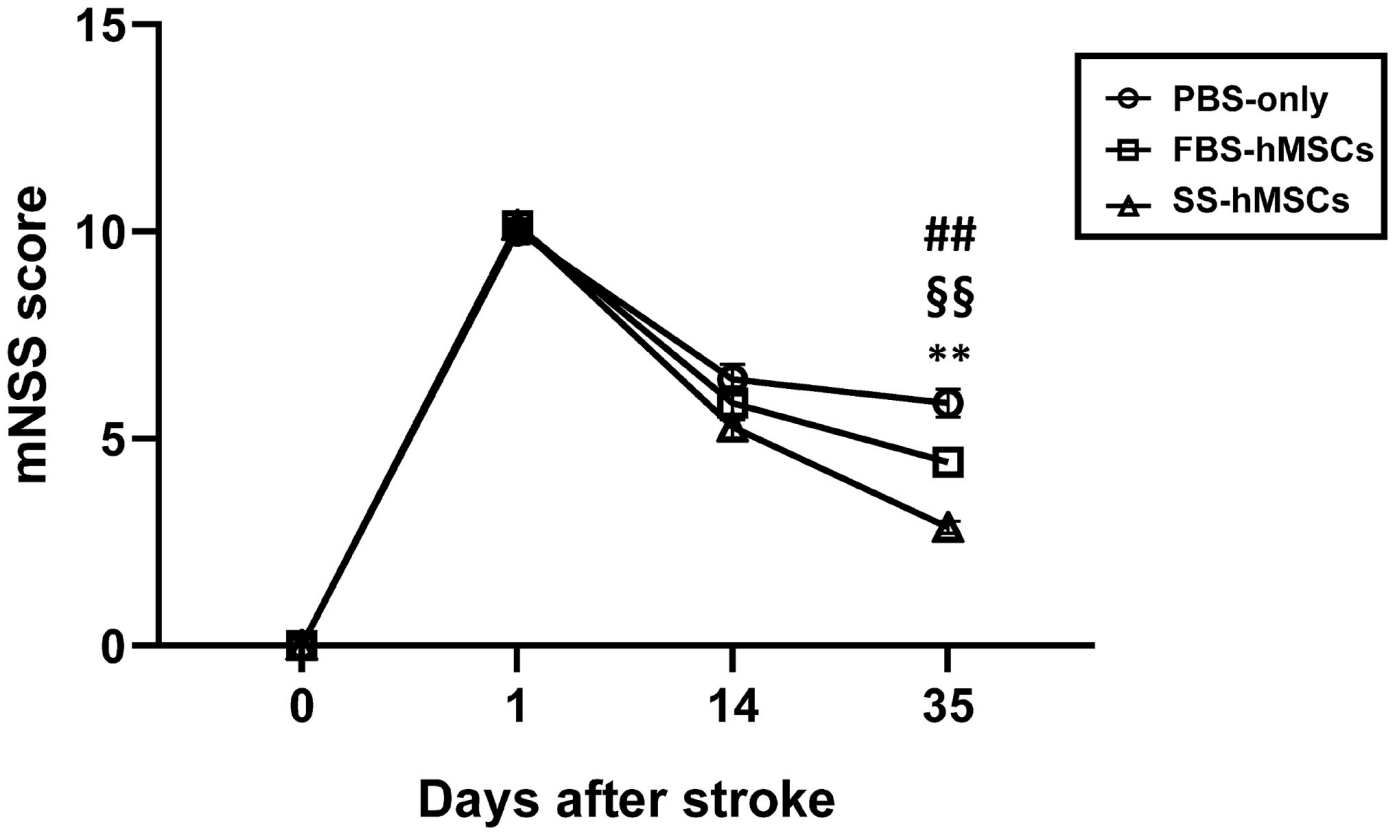
Modified neurological severity score. Functional behavioral improvements were serially assessed in rats treated with PBS-only (n = 6), FBS-hMSCs (n = 6), and SS-hMSCs (n = 6) using modified neurological severity scores (mNSS) (PBS-only vs. SS-hMSCs, **p<0.01; PBS-only vs. FBS-hMSCs, ^##^*p*<0.01; FBS-hMSCs vs. SS-hMSCs, ^§§^*p*<0.01; one-way ANOVA, Tukey post-hoc test).

To examine functional recovery after MSC therapy, behavioral testing was performed serially using mNSS (Fig 2 and S1 Table). In the GEE model, behavioral testing results showed statistically significant interaction between time and treatment (*p*<0.001). At 2 weeks after treatment, there were no significant functional improvement differences between groups, but the SS-hMSCs group showed better trend when compared to the PBS-only group (*p* = 0.087). At 5 weeks after treatment, both the FBS-hMSCs and SS-hMSCs groups showed significant functional improvement compared with the PBS-only groups (*p*<0.01 in both cases). However, the SS-hMSCs group showed significant functional improvement compared with the FBS-hMSCs group (*p* = 0.001).

### Brain morphological changes

**Fig 3:**
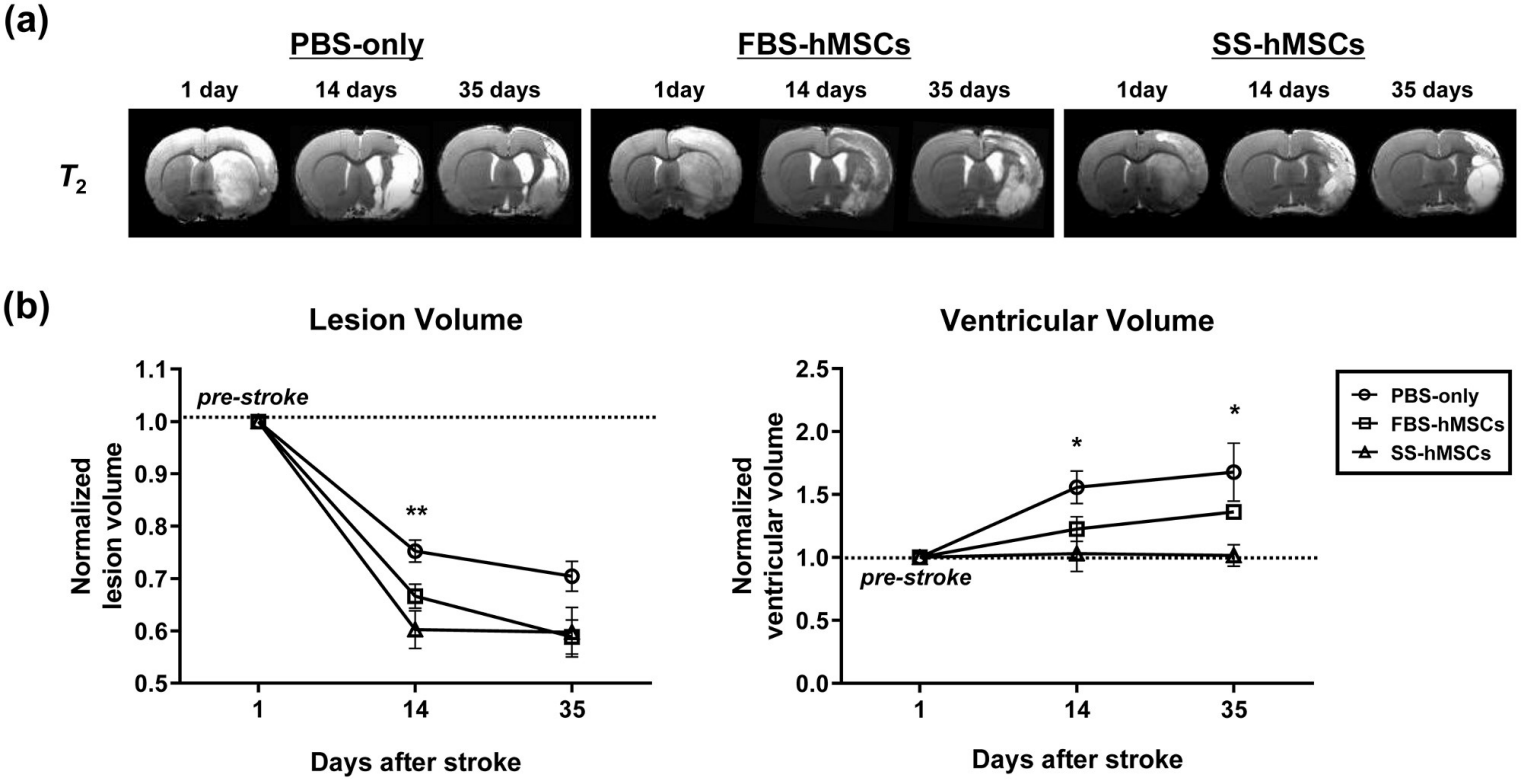
Ischemic lesion and ventricular volume changes. (a) Representative *T*_2_-weighted images of rats treated with PBS-only (n = 6, left), FBS-hMSCs (n = 6, middle), and SS-hMSCs (n = 5, right). (b) Bar graph representing serial changes in ischemic lesion volume (left) and ventricular volume (right) (PBS-only vs. SS-hMSCs, *p<0.05, **p<0.01; one-way ANOVA, Tukey post-hoc test).

To compare the effects of MSC therapy on infarct volume reduction and atrophic changes after stroke, we measured ischemic lesion volume and lateral ventricular volume on *T*_2_-weighted images (Fig 3 and S2 Table). In the GEE model, relative changes of lesional volume and ventricular volume showed statistically significant interaction between time and treatment (*p*<0.001). There were no significant differences in ischemic lesion volumes on day 1 (just before treatment) among the groups (PBS-only vs. FBS-hMSCs, *p* = 0.635; PBS-only vs. SS-hMSCs, *p* = 0.916; FBS-hMSCs vs. SS-hMSCs, *p* = 0.880). Compared to the PBS-only group, the normalized ischemic lesion volume of the SS-hMSCs group was significantly decreased at 2 weeks (*p* = 0.003), but not at 5 weeks (*p* = 0.112). Compared to the PBS-only group, the normalized ischemic lesion volume of the FBS-hMSCs group was numerically decrease at both 2 weeks (*p* = 0.083) and 5 weeks (*p* = 0.081), but statistically insignificant. At day 1 (just before treatment), there was no significant difference in lateral ventricular volumes among the groups (PBS-only vs. FBS-hMSCs, *p* = 0.821; PBS-only vs. SS-hMSCs, *p* = 0.314; FBS-hMSCs vs. SS-hMSCs, *p* = 0.683). Normalized lateral ventricular volume markedly increased in the PBS-only group, and significantly, but to a lesser degree, in the SS-hMSCs group at 2 (*p* = 0.025) and 5 weeks (*p* = 0.04). No significant difference was observed between the PBS-only and FBS-hMSCs groups and the FBS-hMSCs and SS-hMSCs groups, at 2 and 5 weeks (*p*>0.05 for all cases).

### Brain microstructure/connectivity changes

**Fig 4:**
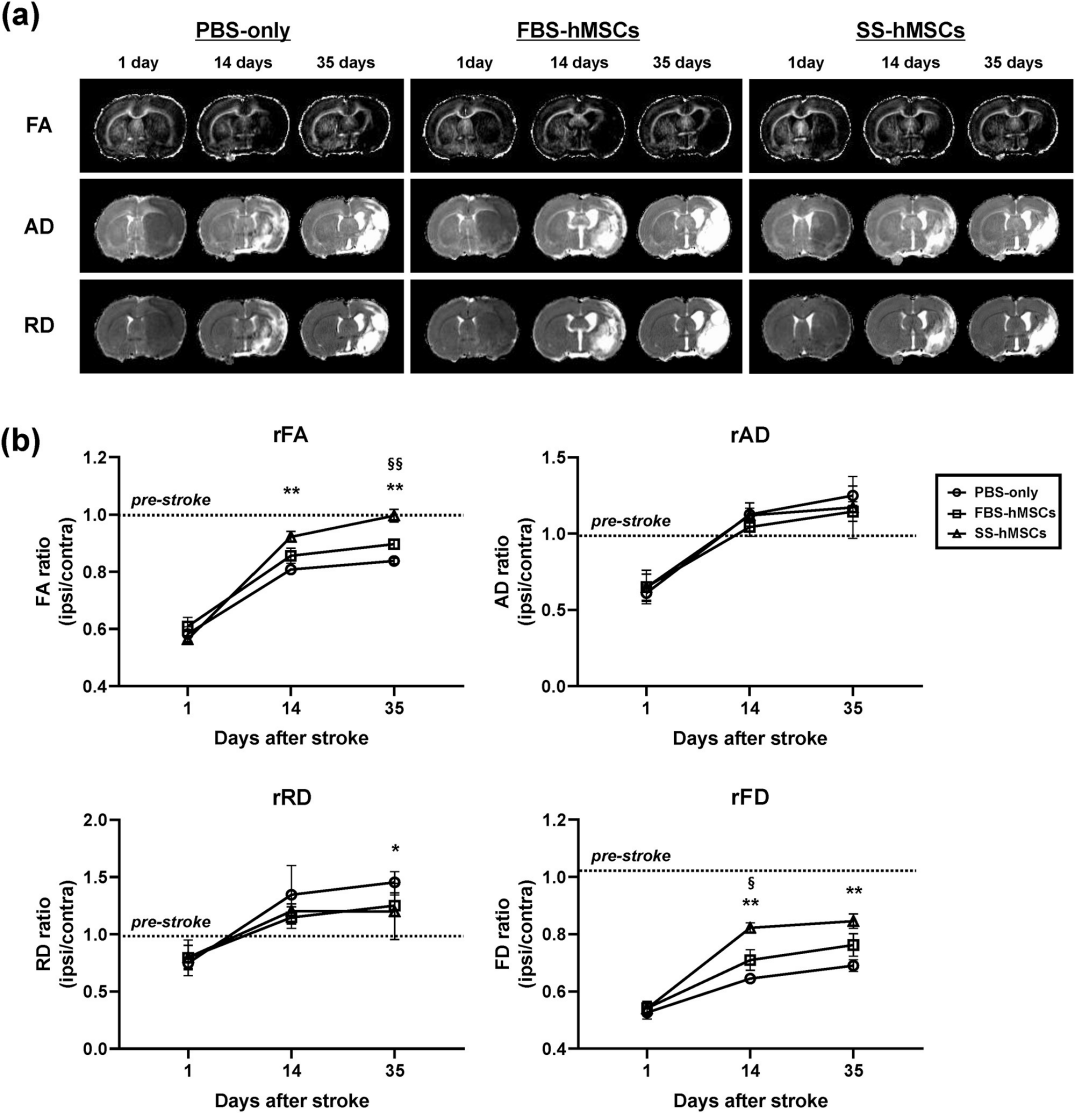
DTI and fiber tractography results. (a) Representative microstructural indices maps of rats treated with PBS-only (n = 6, left), FBS-hMSCs (n = 6, middle), and SS-hMSCs (n = 5, right). (b) Quantitative analysis of relative fractional anisotropy (rFA), relative axial diffusivity (rAD), relative radial diffusivity (rRD), and relative fiber density (rFD) (PBS-only vs. SS-hMSCs, *p<0.05, **p<0.01; FBS-hMSCs vs. SS-hMSCs, ^§^*p*<0.05, ^§§^*p*<0.01; one-way ANOVA, Tukey post-hoc test).

To explore the microstructure/connectivity changes of the ischemic damaged brain after MSCs therapy, quantitative DTI analysis was performed in the ROI at 1 day, and 2 and 5 weeks after tMCAo. Fig 4 and S3 Table demonstrates the representative microstructural indices maps of each group and the relative changes in FA, AD, RD, and FD values calculated for the ischemic recovery area (ischemic recovery area/homologous contralateral area). There were no statistical differences in contralateral absolute FA, AD, RD, and FD values between groups at all follow-up times (p>0.05 for all cases). In the GEE model, relative changes of FA, AD, RD, and FD showed statistically significant interaction between time and treatment (*p*<0.001). In normal rats (n = 6), the relative microstructural indices such as FA, AD, and RD were 1.00 ± 0.03, 0.98 ± 0.02, and 0.98 ± 0.02, respectively. At 1 day after tMCAo, relative FA values of the PBS-only, FBS-hMSCs, and SS-hMSCs groups were 0.58 ± 0.06, 0.61 ± 0.08, and 0.56 ± 0.02, relative AD values were 0.61 ± 0.05, 0.65 ± 0.11, and 0.65 ± 0.09, and relative RD values were 0.75 ± 0.05, 0.79 ± 0.16, and 0.80 ± 0.11, respectively. There were no significant differences of microstructural indices among the groups at 1 day. At 2 weeks, significant differences in the relative FA values were observed between the PBS-only and SS-hMSCs groups (*p* = 0.004), but not between other groups (PBS-only vs. FBS-hMSCs, *p* = 0.221; FBS-hMSCs vs. SS-hMSCs, *p* = 0.090). There were no significant differences of the relative AD and RD values among the groups (p>0.05) at 2 weeks. At 5 weeks, significant differences in the relative FA values were observed between the PBS-only and SS-hMSCs groups (*p*<0.001) and between the FBS-hMSCs and SS-hMSCs groups (*p* = 0.003). The relative FA values of the FBS-hMSCs groups were improved compared to those of the PBS-only group, but did not reach statistical significance (*p* = 0.054). Except only the lower relative RD value of SS-hMSCs groups than PBS-only groups (p = 0.038), there were no significant differences of the relative AD and RD values among the groups at 5 weeks.

**Fig 5:**
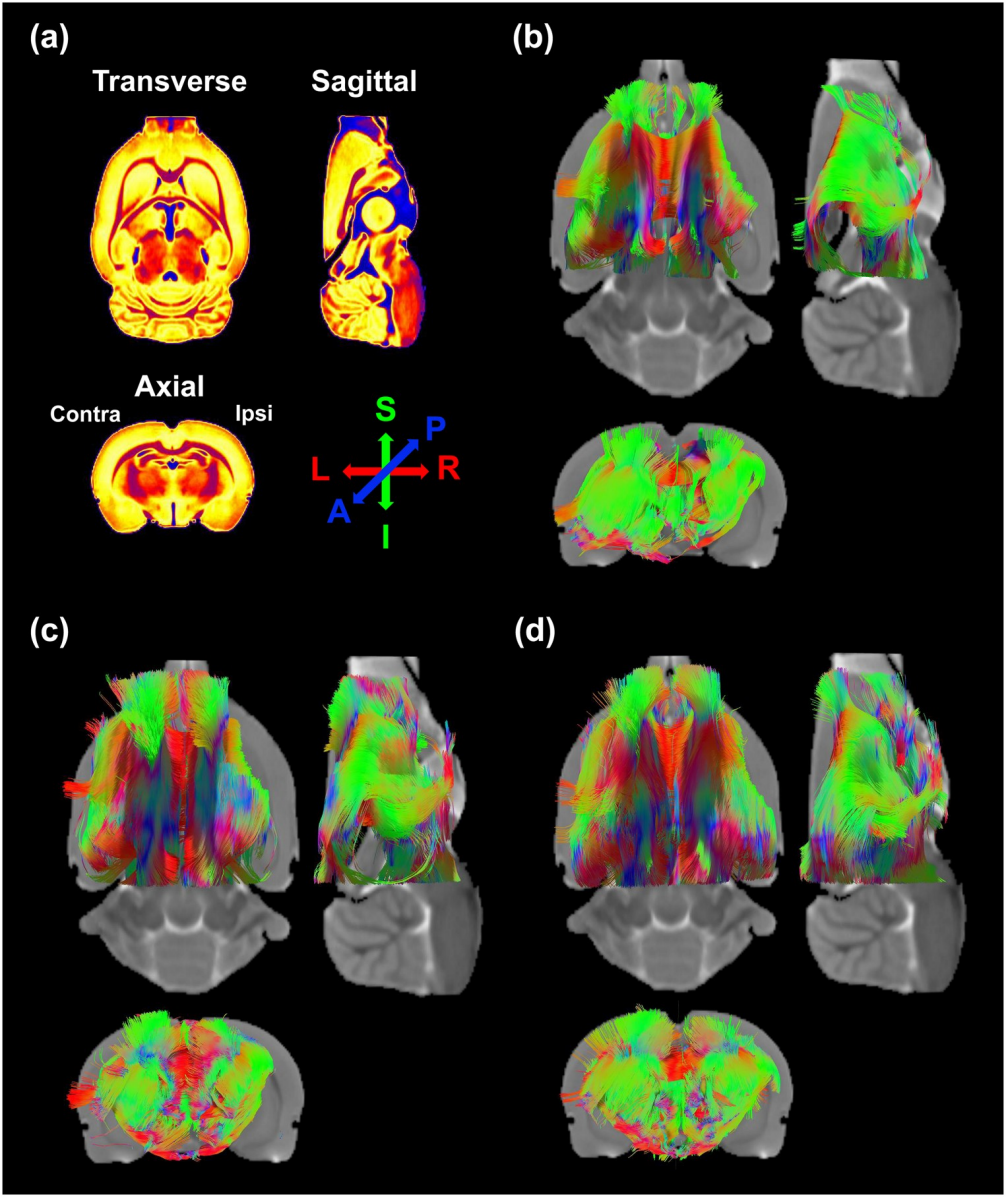
Diffusion tensor tractography images. (a) Imaging plane and color information. Color indicates predominant orientation of reconstructed fiber tracts (red for left-right, green for superior-inferior, and blue for anterior-posterior). Representative fiber tractography from the contra-/ipsilesional ROIs at 5 weeks after treatment with (b) PBS-only, (c) FBS-hMSCs, and (d) SS-hMSCs.

Fig 5 shows representative tractography images in the contra-/ipsilesional ROIs at 5 weeks after stroke. At 1 day after tMCAo, the relative FD values of the PBS-only, FBS-hMSCs, and SS-hMSCs groups were 0.53 ± 0.06, 0.54 ± 0.06, and 0.54 ± 0.04, respectively (1.05 ± 0.02 in non-operated naïve rats, n = 6), and there were no significant differences among the groups. At 2 weeks, significant differences in the relative FD values were observed between the PBS-only and SS-hMSCs groups (*p*<0.001) and between the FBS-hMSCs and SS-hMSCs groups (*p* = 0.015), but not between the PBS-only and FBS-hMSCs groups (*p* = 0.16). At 5 weeks, a significant increase in FD values was observed in the SS-hMSCs group compared to the PBS-only group (*p* = 0.008), but not when comparing the FBS-hMSCs and PBS-only groups (*p* = 0.225).

### Correlation between brain morphological changes and DTI

**Fig 6:**
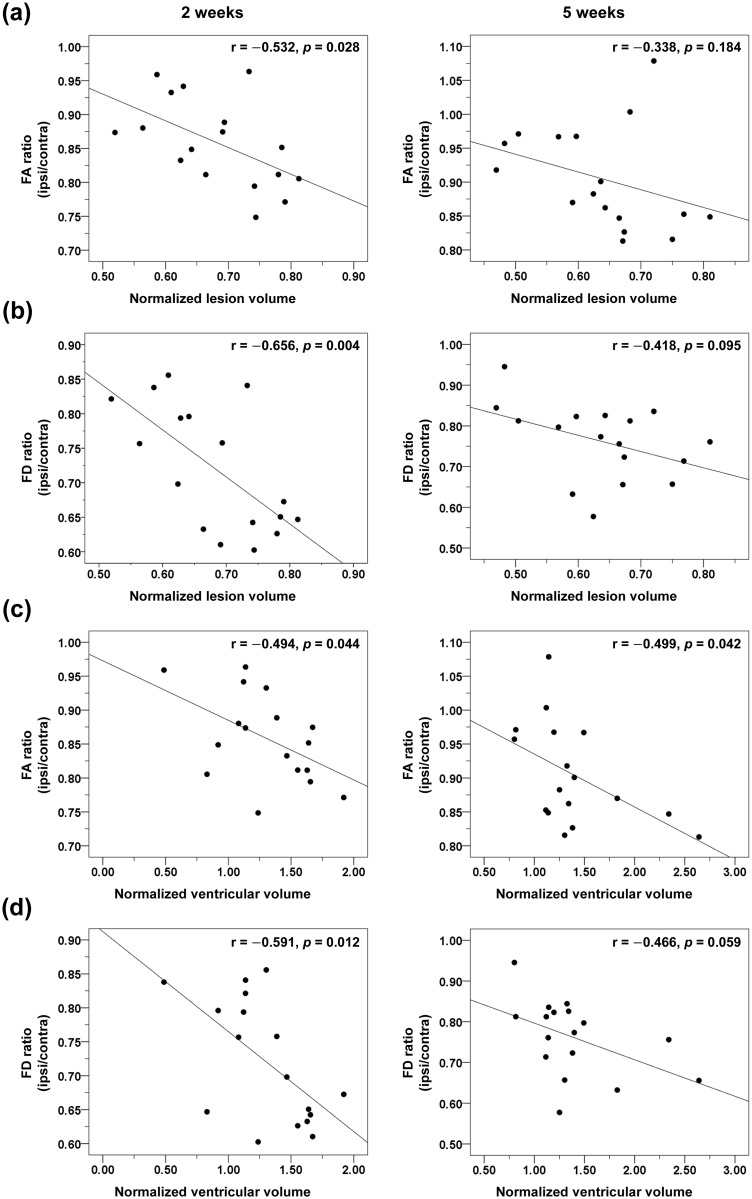
Correlation between *T*_2_-weighted images and DTI findings. Relationship between (a) normalized ischemic lesion volume and fractional anisotropy (FA), (b) normalized ischemic lesion volume and fiber density (FD), (c) normalized ventricular volume and FA, (d) normalized ventricular volume and FD. The left panel shows results at 2 weeks after experimental stroke, and the right panel shows results at 5 weeks. Data are presented as Pearson correlation ratios (r) and *p* values.

To investigate associations between morphological and microstructure/connectivity changes, correlation analyses between *T*_2_-weighted images and DTI findings were performed (Fig 6). At 2 weeks, there was a significant correlation between infarct volume and relative FA (r = −0.532, *p* = 0.028) and FD (r = −0.656, *p* = 0.004) ratios. Similarly, there was a significant correlation between ventricular volume and relative FA (r = −0.494, *p* = 0.044) and FD (r = −0.591, *p* = 0.012) ratios. Such correlations were statistically insignificant at 5 weeks, except for the correlation between relative FA and degree of brain atrophy (r = −0.499, *p* = 0.042). There were no significant correlation between *T*_2_-weighted morphological changes and the relative AD/RD values at both time points (*p*>0.05).

### Histological analysis

**Fig 7:**
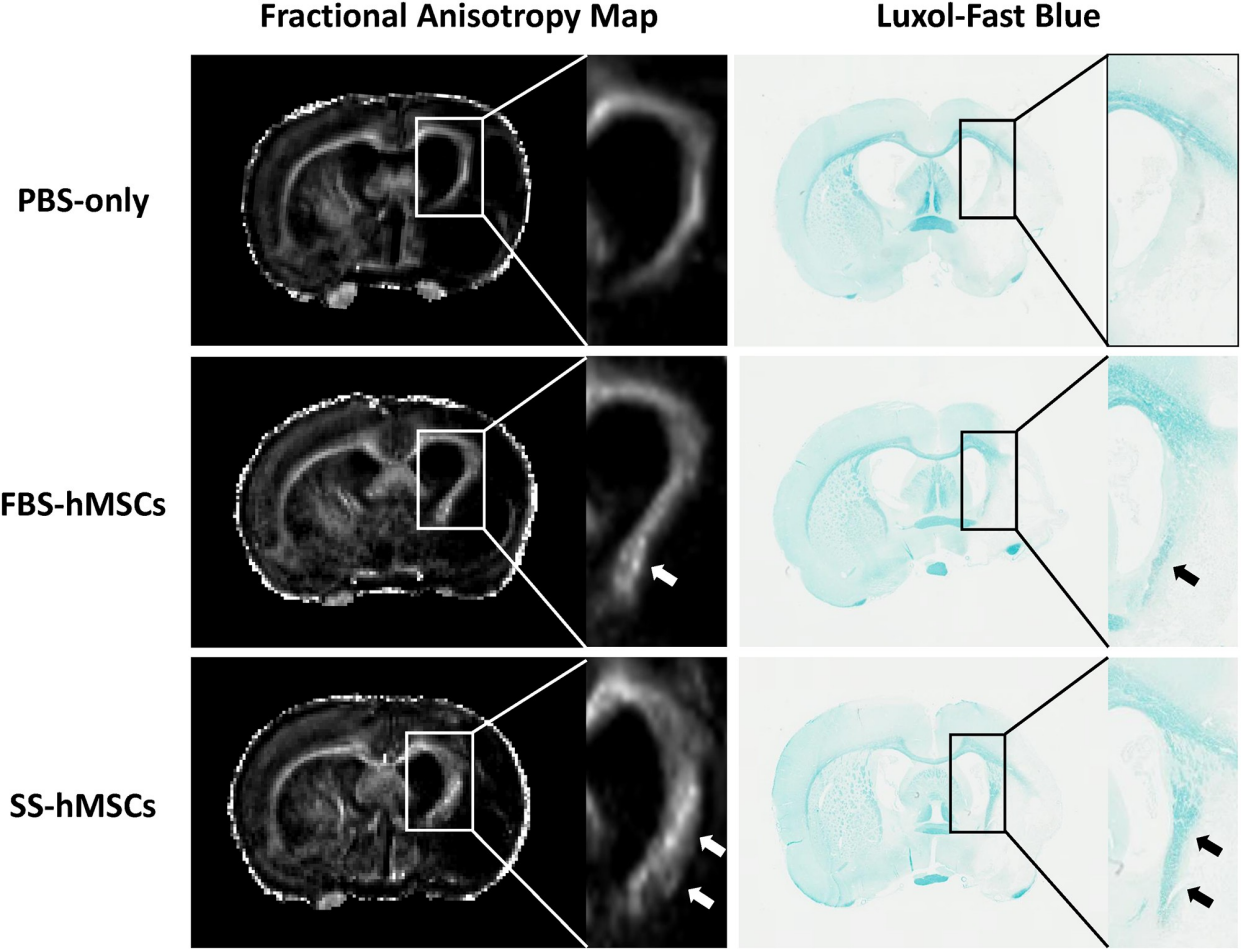
Representative FA maps and immunostaining with LFB. White arrows indicate white matter rearrangement on FA maps, and black arrows indicate the distribution of myelinated fibers.

Five weeks after treatment, immunostaining with LFB for visualization of the myelinated fibers in the ischemic damaged area was performed to test whether MRI results could reflect microstructural alterations after treatment of hMSCs (Fig 7). From FA map, compared to the contralateral side, white matter rearrangement was occurred in the ischemic damaged area of ipsilateral side, and these areas appeared to be more extended in the hMSCs groups than the PBS-only group. The LFB staining showed a larger amount of myelinated fibers in the ischemic damaged region of the SS-hMSCs group than other groups.

## Discussion

The major findings of this study are (a) functional recovery after MSC therapy is associated with morphological and microstructure/connectivity changes on MRI, (b) DTI techniques can be used to compare the effects of two different MSC therapies, and (c) *T*_2_-weighted imaging and DTI features correlate with each other, particularly during the early stage after tMCAo. Our overall approach of using MR imaging data for MSCs therapeutic efficacy monitoring is well taking current STEPS criteria [30]. This might help to provide better correspondence between preclinical and clinical data in the field.

MRI can monitor morphological changes after neurological diseases, noninvasively. In ischemic stroke, infarct volume changes in the acute and chronic stage are important prognostic factors, and lateral ventricular volume dilation is a secondary factor to atrophic changes in the peri-infarct area [31]. Therefore, serial quantitative analysis of these changes, calculated from *T*_2_-weighted imaging, is widely used to detect time dependent restoration or treatment efficacy in ischemic stroke. The present data suggests that morphological changes on *T*_2_-weighted imaging can be used as a sensitive indicator for neurorestoration, and possibly neuroprotection, after ischemic brain damage and stem cell therapy.

In this study, quantitative DTI results were used as an important imaging outcome factor, which were well matched to functional behavioral recovery as measured by mNSS. DTI is a noninvasive imaging technique for visualizing brain microstructural alterations and connectivity, which uses anisotropic diffusion of water molecule [15, 16, 21]. After ischemia, neurorestorative processes such as neurogenesis, angiogenesis and synaptogenesis cause reorganization of neuronal fibers in the peri-infarct area [32–35]. This axonal remodeling increases anisotropic diffusion of water molecules in that microenvironment. The FA value derived from diffusion tensor calculations can indicate the extent of directionality of water diffusion. The increase in FA appears to be correlated with neural fiber tract integrity or gliosis, while a reduction in FA is correlated with functional deficits [12, 14, 22, 36–40]. The other DTI-derived parameters such as AD and RD allow a better understanding of white matter rearrangement after neuronal damage, rather than FA alone. Song and colleagues have demonstrated that RD accurately reflects myelin integrity in mice experimental demyelination model, and Jung and colleagues have suggested that progressive increase of AD and RD at later stages of experimental stroke indicates spontaneous recovery after ischemia [13, 41]. Another DTI derived technique is fiber tractography, which is the only method available for visualizing neural tracts and anatomical connectivity *in vivo*. Reconstructed tracts derived from diffusion tensor tractography show general agreement with histology in post-mortem human and nonhuman animals [42, 43]. In the present study, rats treated with stroke patients’ serum-preconditioned hMSCs showed significant decreases in RD, and increases in FA and reconstructed fiber tracts (Figs 4 and 5). Our previous immunofluorescence staining results [26] showed that MSC therapy increased neurogenesis and angiogenesis in the peri-infarcted area and subventricular zone. Moreover, our present immunostaining results confirmed that a high FA values in the ischemic boundary region of SS-hMSCs treated rats might due to a large number of myelinated fibers. Based on our present histological results, quantitative analysis of DTI-derived microstructural indices and FD can be used as potential imaging biomarkers to identify reorganization of the brain after injury.

In the recovery phase of neurodegenerative diseases, there have been several studies on the relationship between DTI parameters [13, 19, 44]. In the present study, there showed a positive correlation between rFA and rFD (r = 0.818, *p*<0.001 at 2 weeks and r = 0.617, *p* = 0.008 at 5 weeks), and a negative correlation between FA and RD (r = −0.495, *p* = 0.043 at 2 weeks and r = −0.567, *p* = 0.018 at 5 weeks). Our results are in line with the results of previous study which suggested FA as a highly sensitive indicator reflecting axonal integrity [44]. Based on our present immunostaining results with LFB, the recovery mechanism of stem cells after experimental stroke is probably related to both the increase of axonal integrity and distribution of myelinated fibers. However, no significant correlation was observed between FD and RD values, which suggest that unmyelinated fibers or other neural cells may involved in the rearranged white matter tracts. Further study is needed to elucidate the relationship between various DTI parameters and complex recovery mechanisms.

Our analyses showed that DTI parameters were correlated with morphological changes (both infarct volume and atrophic changes) during the early stage of infarct. Conversely, during the later stage of infarct, only the degree of brain atrophy was correlated with DTI parameters. This could be due to the following reasons. First, a marked reduction in infarct volume was observed in all groups, while brain volume/atrophic changes were more consistent. Second, infarct volume may represent neuroprotective effects while brain volume/atrophic changes and DTI may represent neurorestorative effects of stem cells. Up until now, most preclinical studies of stem cell therapy measured the infarct volume at 7–21 days after stroke, and showed conflicting results concerning the degree of reduction of infarct volume [26, 45, 46]. The present study showed that DTI parameters more consistently predicted functional improvement at 5 weeks, than infarct volume. Therefore, it is conceivable that both morphological and microstructure/connectivity images are needed to evaluate the action of stem cells after stroke, which was not performed simultaneously in previous studies.

Various MRI markers are increasingly used to monitor post-stroke recovery after stem cell and pharmacological therapies, in experimental models and humans [47]. Anatomical MRI studies of small animal stroke models have shown conflicting results regarding the effects of stem cell therapy on the reduction of infarct size, and none of the randomized trials of stem cell therapy in stroke patients have compared infarct size between cell therapy groups and controls. Our results are in line with MRI data from our previous clinical study, which showed atrophy within peri-infarct areas and secondary dilations of the adjacent ventricle were less prominent in MSC-treated patients than in control patients [48]. A recent small study showed cavity-filling by new neural tissue formation in patients who received intracerebral injection of a neural stem cell line [49]. Jiang and colleagues used DTI techniques to measure white matter recovery after stem cell therapy in a rat stroke model [22], and Chen and colleagues undertook a stem cell therapy clinical study and showed that DTI fiber number asymmetry scores were correlated with functional recovery, and were reduced in patients who received stem cells [50].

In the present study, two different stem cell therapies, i.e., the FBS-hMSCs and SS-hMSCs groups, were applied to test whether MRI measurements could be used to compare the effects of two different stem cell therapies in a rat model of stroke. Our data showed that treatment with stroke patients’ serum-preconditioned hMSCs (the SS-hMSCs group) is superior to FBS-cultured hMSCs (the FBS-hMSCs group) according to MRI-based assessment, as well as functional behavioral testing. Therefore, multimodal quantitative MRI measurements could be used to monitor the effects of novel cell-based therapy compared to preexisting therapies (e.g., naïve MSCs). The choice of medium is important for the end result of stem cell therapy. Zacharek and colleagues showed that treatment of stroke with bone marrow stem cells obtained from stroke rats resulted in improved functional outcomes, compared to treatment with cells from normal rats, suggesting the possible role of circulating factors on the activation of MSCs in stroke [29]. Our previous preclinical studies showed that culture expansion of MSCs, with serum obtained in the acute phase of stroke, regulated trophic factor release and microRNA profiles, and enhanced recovery after stroke by increasing proliferation rates and decreasing senescence of MSCs, increasing migration to the infarcted brain area, and increasing neurogenesis/angiogenesis [26, 32, 51]. The results of the present preclinical study are in line with a randomized clinical trial [8], and will be confirmed by an ongoing prespecified substudy of recovery MRI [24].

However, caution should be taken when interpreting DTI data. Diffusion tensor modeling is not consistent when voxels contain multiple or crossing fibers, because of the partial volume effect. Also, reconstructed fiber tracts from seeds generally agree with histological data, but regions remote from ROIs could be misinterpreted [43]. Nonetheless, conventional DTI is a simple and promising tool, due to the short acquisition time and ease of processing. In addition, it is well known that DTI-derived microstructural indices, such as FA, AD, and RD values, reflect brain microstructural alterations after damage. In the case of ischemic stroke, neurorestorative processes in the peri-infarct area appear to play an important role in functional recovery after ischemic damage, however, it has not yet been clearly defined how complex these regenerated axonal structures are and what structures are connected to each other. Therefore, we acquired conventional DTI data and performed quantitative analysis of microstructural indices and FD in the ischemic recovered area. Combining high resolution data with advanced diffusion MRI techniques, such as diffusion spectrum magnetic resonance imaging (DSI), more detailed exploration of the complicated recovery mechanisms in ischemic stroke can be carried out. The present study has other limitations. First, correlation between behavioral recovery and MRI measurements was not able to be assessed in individual animals, because previous studies showed that repeated isoflurane-induced inhalational anesthesia for MRI scanning affects the results of behavioral testing [52]. Second, quantitative analysis of immunostaining was not performed in this study. Finally, resting state functional MRI techniques could provide information on functional connectivity in stroke models, which is ongoing in MSC extracellular vesicular therapy studies for small animal models of stroke.

## Conclusion

In conclusion, the results of this study indicate that quantitative measurement of *T*_2_-weighted volume changes and DTI-derived microstructural indices and FD changes well reflect neurological behavioral improvements in rat experimental stroke. Our data suggests that these MRI imaging-based biomarkers could be potential outcome indicators of functional recovery, after stem cell therapy in experimental models and possibly humans.

## Supporting information

S1 Checklist The ARRIVE guidelines checklist. (PDF)

S1 Table Group averaged modified neurological severity scores (mNSS). (DOCX)

S2 Table Group averaged ischemic lesion and ventricular volume (mm^3^). (DOCX)

S3 Table Group averaged relative fractional anisotropy (rFA), relative axial diffusivity (rAD), relative radial diffusivity (rRD), and relative fiber density (rFD) values. (DOCX)
