# Supplementary material for: Brain morphological and connectivity changes on MRI after stem cell therapy in a rat stroke model
Source: PLoS One. 2021 Feb 16;16(2):e0246817. doi: 10.1371/journal.pone.0246817 (PMC7886198; doi:10.1371/journal.pone.0246817)
Supplement: S1 Table — (DOCX) [file pone.0246817.s002.docx]

**S1 Table.**

|  | **0 day** | **1 day** | **14 days** | **35 days** |
| --- | --- | --- | --- | --- |
| **PBS-only** | **0.00 ± 0.00** | **10.00 ± 0.76** | **6.43 ± 0.90** | **5.86 ± 0.83** |
| **FBS-hMSCs** | **0.00 ± 0.00** | **10.14 ± 0.83** | **5.86 ± 0.99** | **4.43 ± 0.49^##^** |
| **SS-hMSCs** | **0.00 ± 0.00** | **10.14 ± 0.83** | **5.29 ± 0.70** | **2.86 ± 0.35^**,^** ^§§^ |

PBS-only vs. SS-hMSCs, ^**^*p*<0.01; PBS-only vs. FBS-hMSCs, ^##^*p*<0.01; FBS-hMSCs vs. SS-hMSCs, ^§§^*p*<0.01; one-way ANOVA, Tukey post-hoc test
