# Supplementary material for: Brain morphological and connectivity changes on MRI after stem cell therapy in a rat stroke model
Source: PLoS One. 2021 Feb 16;16(2):e0246817. doi: 10.1371/journal.pone.0246817 (PMC7886198; doi:10.1371/journal.pone.0246817)
Supplement: S2 Table — (DOCX) [file pone.0246817.s003.docx]

**S2 Table.**

|  | **Infract lesion volume** | | | **Ventricular volume** | | |
| --- | --- | --- | --- | --- | --- | --- |
|  | **1 day** | **14 days** | **35 days** | **1 day** | **14 days** | **35 days** |
| **PBS-only** | **251.94 ± 76.98** | **190.91 ± 66.08** | **181.10 ± 73.07** | **32.00 ± 3.59** | **50.33 ± 13.69** | **54.30 ± 23.07** |
| **FBS-hMSCs** | **290.35 ± 69.94** | **194.24 ± 53.42** | **169.08 ± 36.96** | **34.64 ± 8.18** | **42.07 ± 10.82** | **47.41 ± 12.11** |
| **SS-hMSCs** | **268.61 ± 62.45** | **158.63 ± 22.23^**^** | **158.38 ± 35.02** | **42.18 ± 11.02** | **41.64 ± 13.17^*^** | **41.90 ± 10.62^*^** |

PBS-only vs. SS-hMSCs, ^*^*p*<0.05, ^**^*p*<0.01; one-way ANOVA, Tukey post-hoc test
