# Supplementary material for: Brain morphological and connectivity changes on MRI after stem cell therapy in a rat stroke model
Source: PLoS One. 2021 Feb 16;16(2):e0246817. doi: 10.1371/journal.pone.0246817 (PMC7886198; doi:10.1371/journal.pone.0246817)
Supplement: S3 Table — (DOCX) [file pone.0246817.s004.docx]

**S3 Table.**

|  | **rFA** | | | **rAD** | | | **rRD** | | | **rFD** | | |
| --- | --- | --- | --- | --- | --- | --- | --- | --- | --- | --- | --- | --- |
|  | **1 day** | **14 days** | **35 days** | **1 day** | **14 days** | **35 days** | **1 day** | **14 days** | **35 days** | **1 day** | **14 days** | **35 days** |
| **PBS-only** | **0.58 ± 0.06** | **0.81 ± 0.03** | **0.84 ± 0.02** | **0.61 ± 0.05** | **1.13 ± 0.08** | **1.25 ± 0.06** | **0.75 ± 0.05** | **1.35 ± 0.25** | **1.45 ± 0.09** | **0.53 ± 0.06** | **0.65 ± 0.02** | **0.69 ± 0.05** |
| **FBS-hMSCs** | **0.61 ± 0.08** | **0.86 ± 0.06** | **0.90 ± 0.04** | **0.65 ± 0.11** | **1.04 ± 0.06** | **1.14 ± 0.06** | **0.79 ± 0.16** | **1.15 ± 0.09** | **1.25 ± 0.09** | **0.54 ± 0.06** | **0.71 ± 0.09** | **0.76 ± 0.10** |
| **SS-hMSCs** | **0.56 ± 0.02** | **0.92 ± 0.04^**^** | **1.00 ± 0.05^**,^** ^§§^ | **0.65 ± 0.09** | **1.12 ± 0.05** | **1.17 ± 0.20** | **0.80 ± 0.11** | **1.20 ± 0.06** | **1.20 ± 0.25^*^** | **0.54 ± 0.04** | **0.82 ± 0.04^**,^** ^§^ | **0.85 ± 0.06^**^** |

Mean ionon partegrity PBS-only vs. SS-hMSCs, ^*^*p*<0.05, ^**^*p*<0.01; FBS-hMSCs vs. SS-hMSCs, ^§^*p*<0.05, ^§§^*p*<0.01; one-way ANOVA, Tukey post-hoc test
